# Experimental demonstration of robust entanglement distribution over reciprocal noisy channels assisted by a counter-propagating classical reference light

**DOI:** 10.1038/s41598-017-05008-6

**Published:** 2017-07-06

**Authors:** Rikizo Ikuta, Shota Nozaki, Takashi Yamamoto, Masato Koashi, Nobuyuki Imoto

**Affiliations:** 1 Graduate School of Engineering Science, Osaka University, Toyonaka, Osaka 560-8531 Japan; 2 Photon Science Center, The University of Tokyo, Bunkyo-ku, Tokyo 113-8656 Japan

## Abstract

Embedding a quantum state in a decoherence-free subspace (DFS) formed by multiple photons is one of the promising methods for robust entanglement distribution of photonic states over collective noisy channels. In practice, however, such a scheme suffers from a low efficiency proportional to transmittance of the channel to the power of the number of photons forming the DFS. The use of a counter-propagating coherent pulse can improve the efficiency to scale linearly in the channel transmission, but it achieves only protection against phase noises. Recently, it was theoretically proposed [Phys. Rev. A 87, 052325(2013)] that the protection against bit-flip noises can also be achieved if the channel has a reciprocal property. Here we experimentally demonstrate the proposed scheme to distribute polarization-entangled photon pairs against a general collective noise including the bit flip noise and the phase noise. We observed an efficient sharing rate scaling while keeping a high quality of the distributed entangled state. Furthermore, we show that the method is applicable not only to the entanglement distribution but also to the transmission of arbitrary polarization states of a single photon.

## Introduction

Faithful and efficient distribution of photonic entangled states through noisy and lossy quantum channels is important for realizing various applications of quantum information processing, such as quantum key distribution^1, 2^, quantum repeaters^3^, and quantum computation between distant parties^4, 5^. A decoherence-free subspace (DFS) formed by multiple qubits^6^ is useful to overcome fluctuations during the transmission which cause disturbance on quantum states. So far, a lot of proposals and demonstrations for faithful transmission of photonic quantum states in a DFS against collective noise have been actively studied^7–17^. However, for DFS protocols formed by two or more photons to succeed, all of the photons must arrive at the receiver side, which seriously limits the distribution efficiency of quantum states. When a two-photon DFS is used for faithful quantum communication over a dephasing channel^6, 12^, the transmission rate of quantum states is proportional to *T* ^2^, where *T* is the transmittance of a single photon. When we consider a random unitary (depolarizing) quantum channel, a four-photon DFS is needed to encode a signal photon state^6, 10, 18^, which leads to the transmission rate in the order of *T* ^4^.

The inefficiency of such early DFS schemes has been resolved with an experimental demonstration in the case of entangled photon pairs distributed over a dephasing channel^19^. The key idea to improve the efficiency in the scheme is to prepare a reference single photon for the two-photon DFS from a coherent light pulse with an average photon number of ${\mathcal{O}}({T}^{-1})$ which propagates backwards along the quantum channel from the receiver to the sender of the signal photon. This scheme has a non-zero failure probability of entanglement distribution in the case of *T* = 1, but the scaling of the achieved efficiency of sharing entanglement is proportional to *T* instead of *T* ^2^. While this demonstrated scheme only provides protection against the phase noise, an entanglement distribution scheme against general collective noise with an efficiency proportional to *T* has been recently proposed^20^. This scheme uses the above idea and another key idea^12^ which protects quantum states against general collective noise by using the two-photon DFS against collective dephasing noise at the price of using two communication channels and receiving a constant loss. Such state protection is provided by the reciprocity of the quantum channel and a property of quantum entanglement that disturbance on one half of an entangled system is equivalent to disturbance on the other half. Optical fibres are known to be reciprocal media, and thus an experimental proof of the protection method is important for efficient fibre-based long-distance quantum communication.

In this paper, we for the first time report an experimental demonstration with an efficiency proportional to *T* against collective noise including not only phase noise but also bit flip noise by using the proposed method^20^. We also show that our method is applicable to the distribution of any single-photon quantum state with the use of the quantum parity check^21^.

## Results

### Theory

**Figure 1:**
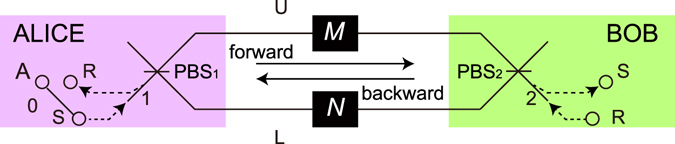
Entanglement distribution protocol by using a counter-propagating photon as a reference light^20^. When photons S and R are reached at port 2 and 1, respectively, the entangled photon pair between Alice and Bob is extracted by the quantum parity check on photons A and R.

We first review the protocol for sharing an entangled photon pair against general collective noise^20^, in which it is assumed that the party is allowed to use two noisy channels. The conceptual setup of the protocol is shown in Fig. 1. First the sender Alice prepares a maximally entangled photon pair A and S as ${|{\varphi }^{+}\rangle }^{{\rm{AS}}}\equiv$ $({|{\rm{H}}\rangle }^{{\rm{A}}}{|{\rm{H}}\rangle }^{{\rm{S}}}+{|{\rm{V}}\rangle }^{{\rm{A}}}{|{\rm{V}}\rangle }^{{\rm{S}}})/\sqrt{2}$, and sends the signal photon S to Bob after injecting the photon to a polarizing beamsplitter (PBS_1_) connected to two quantum channels from port 1. Here |H〉 and |V〉 represent horizontal (H) and vertical (V) polarization states of a photon, respectively. On the other hand, the receiver Bob prepares a reference photon R in the state ${|{\rm{D}}\rangle }^{{\rm{R}}}\equiv ({|{\rm{H}}\rangle }^{{\rm{R}}}+{|{\rm{V}}\rangle }^{{\rm{R}}})/\sqrt{2}$, and sends it to Alice after injecting the photon to another PBS (PBS_2_) from port 2.

At the noisy quantum channels, unknown polarization transformations are added on the photons. For the forward (backward) propagation from Alice (Bob) to Bob (Alice), we denote the linear operators of lossy linear optical media for upper (U) and lower (L) optical paths in Fig. 1 by *M*_f(b)_ and *N*_f(b)_, respectively. The subscripts f and b indicate the forward and backward directions, respectively. The media satisfy $0\le \langle v|{M}_{{\rm{f}}({\rm{b}})}^{\dagger }{M}_{{\rm{f}}({\rm{b}})}|v\rangle \le 1$ and $0\le \langle v|{N}_{{\rm{f}}({\rm{b}})}^{\dagger }{N}_{{\rm{f}}({\rm{b}})}|v\rangle \le 1$ for any state |*v*〉. We assume that the media satisfy the property called reciprocity. As is explained in the previous paper^20^, for counter-propagating light pulses through any reciprocal media including the situation considered in this paper, Alice and Bob can choose their coordinate systems such that the action of the operators on the single photon satisfies

1 $$\langle i|{{\rm{\Omega }}}_{{\rm{b}}}|j\rangle =\langle j|Z{{\rm{\Omega }}}_{{\rm{f}}}Z|i\rangle $$

for Ω = *M*, *N* and *i*, *j* ∈ {H, V}, where *Z* = |H〉〈H| − |V〉〈V|. In the paper, we chose the coordinate systems satisfying Eq. ((1)).

After the transmission through the channels, the separated components of the photons S and R are recombined at PBS_2_ and PBS_1_, respectively. Alice and Bob postselect the events of the photon S coming from port 2 and the photon R coming from port 1. As a result, the initial state |*ϕ* ^+^〉^AS^|D〉^R^ is transformed as

2 $$\begin{array}{ccc}{|{\varphi }^{+}\rangle }^{{\rm{A}}{\rm{S}}}{|{\rm{D}}\rangle }^{{\rm{R}}} & \to  & ({|{\rm{H}}\rangle }_{0}^{{\rm{A}}}\otimes {M}_{{\rm{f}}}{|{\rm{H}}\rangle }_{1}^{{\rm{S}}}\otimes {M}_{{\rm{b}}}{|{\rm{H}}\rangle }_{2}^{{\rm{R}}}\\  &  & +{|{\rm{H}}\rangle }_{0}^{{\rm{A}}}\otimes {M}_{{\rm{f}}}{|{\rm{H}}\rangle }_{1}^{{\rm{S}}}\otimes {N}_{{\rm{b}}}{|{\rm{V}}\rangle }_{2}^{{\rm{R}}}\\  &  & +{|{\rm{V}}\rangle }_{0}^{{\rm{A}}}\otimes {N}_{{\rm{f}}}{|{\rm{V}}\rangle }_{1}^{{\rm{S}}}\otimes {M}_{{\rm{b}}}{|{\rm{H}}\rangle }_{2}^{{\rm{R}}}\\  &  & +{|{\rm{V}}\rangle }_{0}^{{\rm{A}}}\otimes {N}_{{\rm{f}}}{|{\rm{V}}\rangle }_{1}^{{\rm{S}}}\otimes {N}_{{\rm{b}}}{|{\rm{V}}\rangle }_{2}^{{\rm{R}}})/2\end{array}$$

3 $$\begin{array}{lll} & \to  & (\langle {\rm{H}}|{M}_{{\rm{f}}}|{\rm{H}}\rangle \langle {\rm{H}}|{M}_{{\rm{b}}}|{\rm{H}}\rangle {|{\rm{H}}\rangle }_{0}^{{\rm{A}}}{|{\rm{H}}\rangle }_{2}^{{\rm{S}}}{|{\rm{H}}\rangle }_{1}^{{\rm{R}}}\\  &  & +\langle {\rm{H}}|{M}_{{\rm{f}}}|{\rm{H}}\rangle \langle {\rm{V}}|{N}_{{\rm{b}}}|{\rm{V}}\rangle {|{\rm{H}}\rangle }_{0}^{{\rm{A}}}{|{\rm{H}}\rangle }_{2}^{{\rm{S}}}{|{\rm{V}}\rangle }_{1}^{{\rm{R}}}\\  &  & +\langle {\rm{V}}|{N}_{{\rm{f}}}|{\rm{V}}\rangle \langle {\rm{H}}|{M}_{{\rm{b}}}|{\rm{H}}\rangle {|{\rm{V}}\rangle }_{0}^{{\rm{A}}}{|{\rm{V}}\rangle }_{2}^{{\rm{S}}}{|{\rm{H}}\rangle }_{1}^{{\rm{R}}}\\  &  & +\langle {\rm{V}}|{N}_{{\rm{f}}}|{\rm{V}}\rangle \langle {\rm{V}}|{N}_{{\rm{b}}}|{\rm{V}}\rangle {|{\rm{V}}\rangle }_{0}^{{\rm{A}}}{|{\rm{V}}\rangle }_{2}^{{\rm{S}}}{|{\rm{V}}\rangle }_{1}^{{\rm{R}}})\mathrm{/2}.\end{array}$$

The subscripts of the state vectors represent the spatial modes of the photons. The second arrow shows the postselection of spatial modes of photons S and R after transformation at PBS_2_ and PBS_1_. From Eq. (1), we see that the coefficients of the second and the third terms in Eq. (3) are the same. Alice can extract the two terms by using the quantum parity check^21^ on the photons A and R. This is done by injecting the photons A and R into the two input ports of a PBS. Under the assumption that only the cases where each output port has at least one photon are post-selected, the action of the PBS is described by ${|{\rm{H}}\rangle }_{0}{|{\rm{V}}\rangle }_{1}{\langle {\rm{H}}|}_{0}^{{\rm{A}}}{\langle {\rm{V}}|}_{1}^{{\rm{R}}}+{|{\rm{V}}\rangle }_{0}{|{\rm{H}}\rangle }_{1}{\langle {\rm{V}}|}_{0}^{{\rm{A}}}{\langle {\rm{H}}|}_{1}^{{\rm{R}}}$. Then she performs a projective measurement $\{{|{\rm{D}}\rangle }_{1},|{\bar{{\rm{D}}}}_{1}\rangle \}$ on the photon in output port 1, where $|\bar{{\rm{D}}}\rangle \equiv (|{\rm{H}}\rangle -|{\rm{V}}\rangle )/\sqrt{2}$. Thus, by performing a proper phase compensation after the projection, a maximally entangled state $({|{\rm{H}}\rangle }_{0}{|{\rm{H}}\rangle }_{2}^{{\rm{S}}}+{|{\rm{V}}\rangle }_{0}{|{\rm{V}}\rangle }_{2}^{{\rm{S}}})/\sqrt{2}$ is extracted. The success probability of the protocol is given by ${|\langle {\rm{H}}|{M}_{{\rm{f}}}|{\rm{H}}\rangle \langle {\rm{V}}|{N}_{{\rm{f}}}|{\rm{V}}\rangle |}^{2}\mathrm{/2}$. In our experiment, we construct the lossy and noisy channels by ${M}_{{\rm{f}}({\rm{b}})}=\sqrt{T}{U}_{{\rm{f}}({\rm{b}})}^{{\rm{U}}}$ and ${N}_{{\rm{f}}({\rm{b}})}=\sqrt{T}{U}_{{\rm{f}}({\rm{b}})}^{{\rm{L}}}$, where ${U}_{{\rm{f}}({\rm{b}})}^{{\rm{U}}}$ and ${U}_{{\rm{f}}({\rm{b}})}^{{\rm{L}}}$ are unitary operators, and *T* is a transmittance of an identical polarization-independent linear loss component. Since the two channels are independent, the success probability becomes *T* ^2^ *T*_U_ *T*_L_/2, where *T*_U_ and *T*_L_ are given by the average values of ${|\langle {\rm{H}}|{U}_{{\rm{f}}}^{{\rm{U}}}|{\rm{H}}\rangle |}^{2}$ about ${U}_{{\rm{f}}}^{{\rm{U}}}$ and ${|\langle {\rm{V}}|{U}_{{\rm{f}}}^{{\rm{L}}}|{\rm{V}}\rangle |}^{2}$ about ${U}_{{\rm{f}}}^{{\rm{L}}}$, respectively. If ${U}_{{\rm{f}}}^{{\rm{U}}}$ and ${U}_{{\rm{f}}}^{{\rm{L}}}$ are completely random, we obtain *T*_U_ = *T*_L_ = 1/2. In the experiment, while we switch the unitary operators discretely as described later, the transmission is kept to *T*_U_ = *T*_L_ = 1/2, resulting in the success probability of *T* ^2^/8.

The efficiency ${\mathcal{O}}({T}^{2})$ of sharing the entangled states is improved to ${\mathcal{O}}(T)$ by using a coherent light pulse instead of using the single photon for R. Suppose that an average photon number of the coherent light received by Alice is *μ*, which means that Bob prepares the coherent light of average photon number *μT* ^−1^. Since the quantum channel considered in this paper is a linear optical channel, the protocol with the use of the coherent light for R works well as was described above when Alice receives one photon in the pulse R and Bob receives the signal photon S, which occurs at a probability of *c*_1_ *μT*, where *c*_1_ is a coefficient reflecting local losses such as those in encoding and decoding, and it is constant independent of *μ* and *T*. Unfortunately, a conventional quantum parity check with linear optics and threshold photon detectors at Alice’s side cannot perfectly discard the cases with multiple photons received in the pulse R. Such unwanted events occur at a probability of *c*_2_ *μ* ^2^ *T*, where *c*_2_ is also a constant, and they degrade the fidelity of the final state. As a result, by choosing the value of *μ* independent of *T* such that ${c}_{1}\gg {c}_{2}\mu$ is satisfied, which ensures that the probability of the unwanted events being sufficiently small, a high-fidelity entangled photon pair is shared with an overall success probability *c*_1_ *μT* which is proportional to *T*.

### Experimental setup

**Figure 2:**
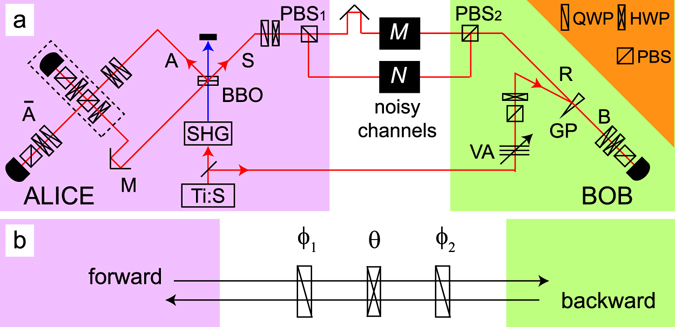
(**a**) Our experimental setup. The component surrounded by a broken line is the quantum parity check and projection onto |D〉 for extracting and decoding the DFS. QWPs, HWPs and PBSs installed into paths $\overline{{\rm{A}}}$ and B are for the quantum state tomography of the distributed states. Additional QWPs and HWPs in paths A and S at Alice’s side are for the quantum state tomography of the initial photon pair. (**b**) The reciprocal noisy channel denoted by *M* or *N* in Fig. 2(a). The operation is *Q*(*ϕ*_2_)*H*(*θ*)*Q*(*ϕ*_1_) for the forward-propagating photons, and is *Q*(−*ϕ*_1_)*H*(−*θ*)*Q*(−*ϕ*_2_) for the backward-propagating ones.

The experimental setup is shown in Fig. 2(a). Light from a mode-locked Ti:sapphire (Ti:S) laser (wavelength: 790 nm; pulse width: 100 fs; repetition rate: 80 MHz) is divided into two beams. One beam is used at Alice’s side. It is frequency doubled (wavelength: 395 nm; power: 75 mW) by second harmonic generation (SHG) for preparing |*ϕ* ^+^〉^AS^ through spontaneous parametric down conversion (SPDC) by a pair of type-I and 1.5-mm-thick *β*-barium borate (BBO) crystals. The photon pair generation probability per pulse is *γ* ≈ 2 × 10^−3^. Photon S enters the two noisy channels after PBS_1_. After passing through the noisy channels, the H- and V-polarized components are extracted and recombined by PBS_2_, and propagate to a photon detector in mode B after a wedged glass plate (GP) whose reflectance is less than 10%. The other beam from the Ti:S laser is used to prepare a coherent light as a reference light R at Bob’s side. The intensity of the coherent light is adjusted by a variable attenuator (VA) in such a way that *μ* ≈ 0.9 × 10^−1^ when it arrives at Alice’s side after PBS_1_. The polarization of the coherent light is set to the diagonal polarization by a PBS and a half wave plate (HWP) after VA. The coherent light R is reflected by GP and then propagates along the noisy channels after PBS_2_ along the same spatial paths as photon S.

After the transmission of photons through the quantum channels, Alice performs the quantum parity check and the projection for extracting the DFS and decoding to a qubit state of a single photon, which is shown in the broken box in Fig. 2(a). After the reference light pulse R passes through the first HWP of the quantum parity check which flips H (V) to V (H), Alice mixes the light pulses A and R at the first PBS with a temporal delay adjusted by mirrors (M). Then she projects the photons coming from one of the output ports of the first PBS onto the diagonal polarization by using the second HWP and the second PBS of the quantum parity check. Alice postselects the cases where at least one photon is detected in each of the output ports of the PBS. On the other hand, Bob postselects the cases where at least one photon is received in the pulse S and hence it appears in mode B. Under this post-selection rule, Alice and Bob share the photon pairs in modes $\overline{{\rm{A}}}$ and B which are in state ${|{\varphi }^{+}\rangle }_{\overline{{\rm{A}}}{\rm{B}}}$ ideally. All detectors are silicon avalanche photon detectors which are coupled to single-mode optical fibres after spectral filtering with a bandwidth of 2.7 nm.

In this experiment, we simulate a lossy depolarizing quantum channel for each noisy channel. The channel is composed of one HWP sandwiched by two quarter wave plates (QWPs) as shown in Fig. 2(b). The operations of a HWP and a QWP acting on a single photon are described by $H(\theta )=\,\cos (2\theta )Z-\,\sin (2\theta )X$ and $Q(\varphi )=(iI-\,\cos (2\varphi )Z+\,\sin (2\varphi )X)/\sqrt{2}$, respectively^22^, where *I* = |H〉〈H| + |V〉〈V|, *X* = |*H*〉〈*V*| + |*V*〉〈*H*| and *Y* = −*i*|*H*〉〈*V*| + *i*|*V*〉〈*H*|. Here *θ* and *ϕ* are rotation angles of the wave plates. The operation *Q*(*ϕ*_2_)*H*(*θ*)*Q*(*ϕ*_1_) on forward-propagating photons works as *I*, *X*, *Y* and *Z* for the settings of (*ϕ*_1_, *θ*, *ϕ*_2_) = (0, 0, 0), (0, −*π*/4, 0), (*π*/2, −*π*/4, 0) and (*π*/4, 0, *π*/4), respectively, up to global phases. For the backward-propagating photons, the operation of the channel becomes *Q*(−*ϕ*_1_)*H*(−*θ*)*Q*(−*ϕ*_2_). This results in the operation of the backward channel as *I*, *X*, *Y* and *Z* for the above four angle settings. For simulating the two depolarizing channels, we slowly switched among the four settings of the wave plates independently in the two noisy channels. In order to introduce photon loss, we insert identical neutral density (ND) filters in the two channels.

### Experimental results

We first performed the quantum state tomography^22^ of the initial photon pair in modes A and S prepared by the SPDC. We reconstructed the density operator *ρ*_AS_ of the two-photon state with the use of the iterative maximum likelihood method^23^. The observed fidelity to the maximally entangled state |*ϕ* ^+^〉_AS_ defined by *F* = 〈*ϕ* ^+^|*ρ*_AS_|*ϕ* ^+^〉 was *F* = 0.97 ± 0.01, the purity defined by $P={\rm{tr}}({\rho }_{{\rm{AS}}}^{2})$ was *P* = 0.95 ± 0.02, and entanglement of formation (EoF) *E* ^24^ was *E* = 0.93 ± 0.02. This result shows Alice prepares a highly entangled photon pair. The count rate of the two-photon state was about 2.4 kHz.

**Figure 3:**
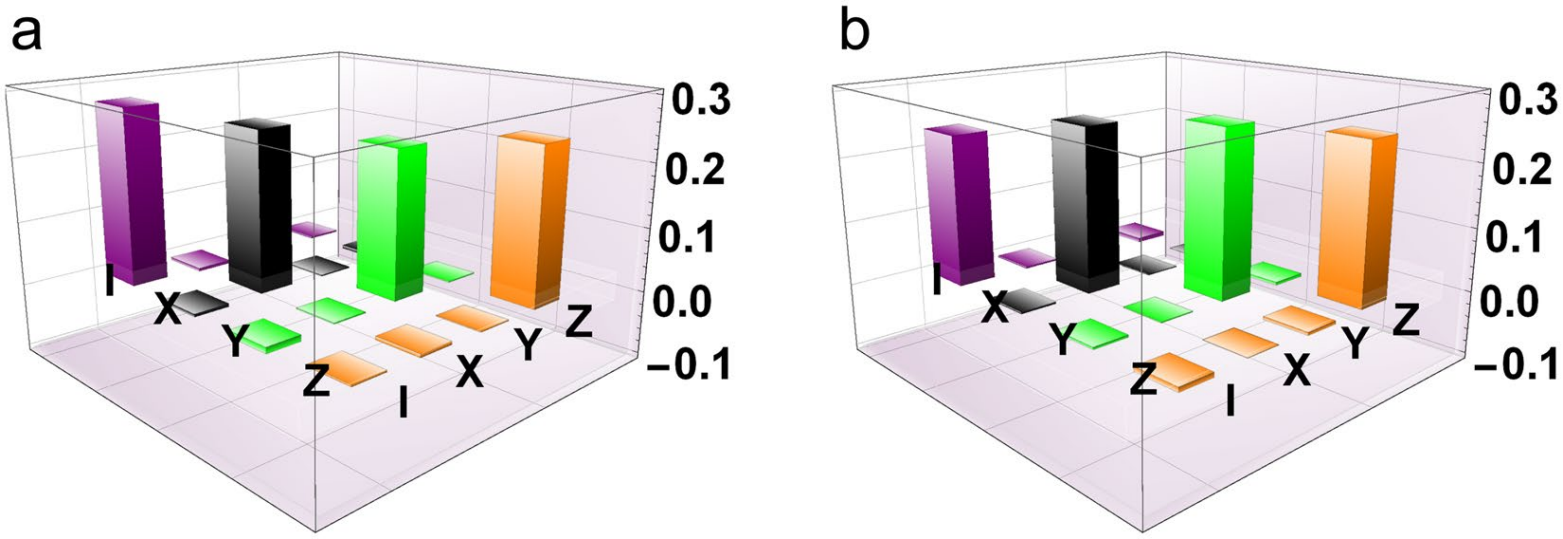
The real part of the reconstructed process matrix. (**a**) For the forward propagation. (**b**) For the backward propagation.

Before we perform the entanglement distribution by the DFS, we performed the process tomography^25^ of the noisy channel composed of the three wave plates for forward and backward propagation of photons. For this, we sent the photon S entangled with photon A to the noisy channel with the forward and backward configuration, and then we performed state tomography of the two-photon output state. When we sequentially switch the angles of the wave plates for simulating *I*, *X*, *Y* and *Z*, the process matrices of the channel are reconstructed as shown in Fig. 3(a,b), by assuming that the initial state is the perfect entangled state |*ϕ* ^+^〉_AS_. The ideal process matrix of the depolarizing channel is a diagonal matrix with all entries being 0.25 because the channel is the equally-randomized operation of *I*, *X*, *Y* and *Z*. From Fig. 3(a,b), we see that the quantum channel well simulates the depolarizing channel for both directions.

**Figure 4:**
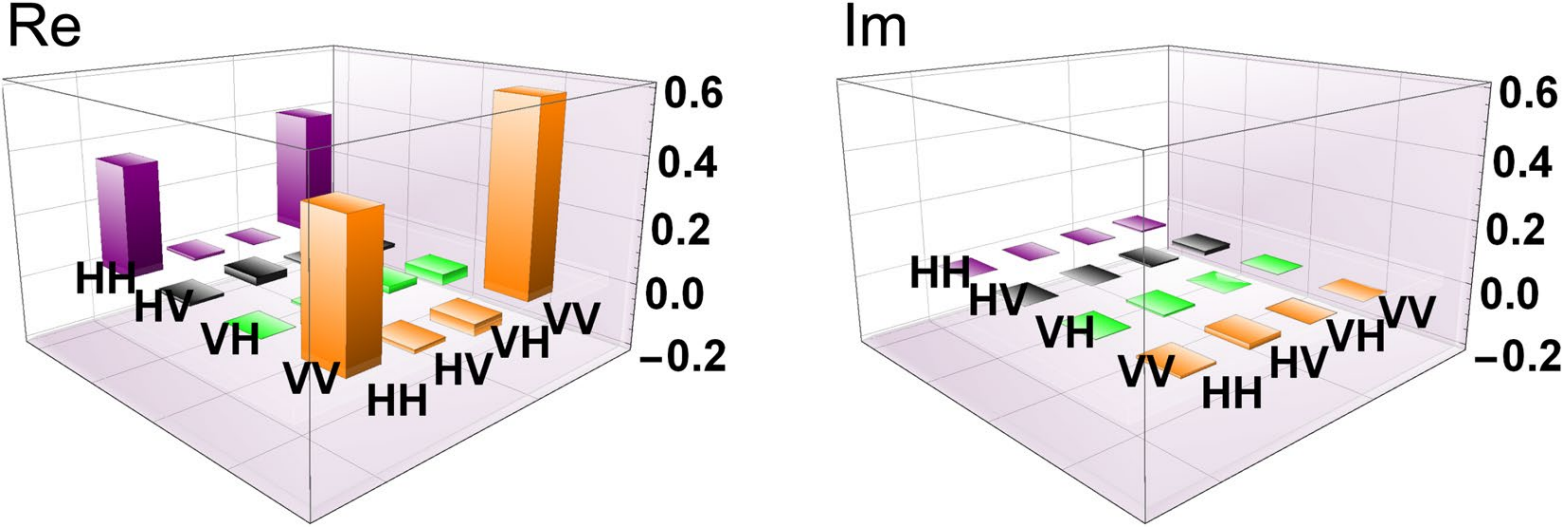
The reconstructed density matrix *ρ*_AB_ for *T* = 1.

**Figure 5:**
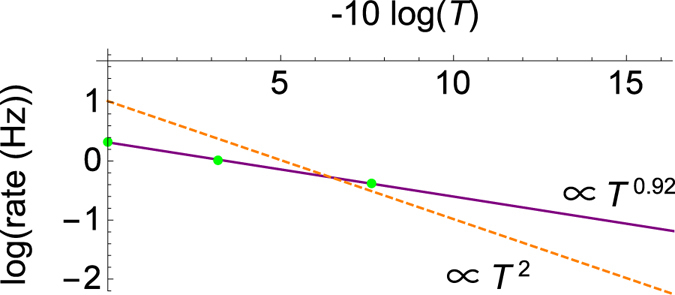
The observed count rate of the states protected by the DFS. The solid line fitted to the experimental data proportional to *T* ^0.92±0.02^. The broken line proportional to *T* ^2^ is expected when the 2-qubit DFS method with a forward-propagating reference single photon^12^ is used. We assumed that the encoding and decoding are performed by using a conventional linear optics^12^. The line passes through a value *μ* ^−1^/2 times as large as the observed rate for *T* = 1. We note that the standard deviations of all data with an assumption of the Poisson statistics of the counts are much smaller than the size of the green circles.

Next, we performed our DFS scheme. When we inserted no ND filters to the channels which is regarded as the case of transmittance *T* = 1, the reconstructed density operator of the photon pair shared between Alice and Bob were shown in Fig. 4. The fidelity, the purity and EoF of *ρ*_AB_ are *F* = 0.89 ± 0.02, *P* = 0.83 ± 0.03 and *E* = 0.69 ± 0.06, respectively. The result shows that the DFS scheme protects the entanglement against collective depolarizing noise. The count rate of the two-photon state was about 2 Hz. This value is consistent with *μη*/16 obtained by considering the ideal success probability *T* ^2^/8 for *T* = 1 of this protocol when a single photon is used for an ancillary qubit, 1/2 loss at the projection after the quantum parity check in this setup, and the effect of using the coherent light with *μη* ≈ 0.01, where *η* is a quantum efficiency of the detectors at Alice’s side. When we inserted ND filters to the channels for *T* to be ≈0.48 and ≈0.17, in order for *μ* to be a constant at Alice’s side, we chose the intensity of the reference light pulse R at Bob’s side to be *T* ^−1^ times as high as that for the case of *T* = 1. The observed fidelity, the purity and EoF of the reconstructed state for each transmittance are shown in Table 1. For all *T*, the highly entangled photon pairs were shared between Alice and Bob. The sharing rate of the final states for each *T* is shown in Fig. 5, which shows that the sharing rate is proportional to *T*.

**Table 1:** The experimental results of the fidelity *F*, purity *P* and EoF *E* of the reconstructed density operators distributed by our scheme for each transmittance *T*.

*T*	Fidelity (*F*)	Purity (*P*)	EoF (*E*)	Fidelity^th^
1	0.89 ± 0.02	0.83 ± 0.03	0.69 ± 0.06	${0.88}_{-0.02}^{+0.01}$
0.48	0.85 ± 0.02	0.79 ± 0.03	0.63 ± 0.06	0.86 ± 0.02
0.17	0.87 ± 0.02	0.80 ± 0.04	0.66 ± 0.06	${0.92}_{-0.02}^{+0.03}$

Fidelity^th^ is the value predicted by the theoretical model with the experimental parameters, in which the error bars come from the observed values of the visibility between the light pulses at Alice’s side.

We consider the reason for the degradation of the entanglement. For this, we constructed a theoretical model in the same manner as discussed in the paper^19^. In the model, we assume that each pulse is in a single mode but includes multiple photons, and we take into account mode matching *V*_sp_ between mode A and mode R. For calculating the fidelities in this model, we used the experimental parameters *γ* ≈ 2 × 10^−3^, *μη* ≈ 0.01, *η* ≈ 0.13, *η*_b_ ≈ 0.11, *d* ≈ 1.3 × 10^−6^, where *η*_b_ is the quantum efficiency of the detector at Bob’s side, and *d* is the dark count rate per pulse. *V*_sp_ was determined via the observation of an HOM dip between modes A and R. In the Discussion part, we will show an example of the HOM dip. Since the depth of the dip can be predicted within the same model, comparison to the observed value gives an estimate of *V*_sp_. The estimation was done separately for the three values of transmission *T*. The obtained values of *V*_sp_ are 0.89 ± 0.03, 0.86 ± 0.04 and ${0.99}_{-0.05}^{+0.01}$ for *T* = 1, 0.48 and 0.17, respectively. Fidelities predicted by the theory are shown in Table 1. They are in good agreement with the observed values. In the model, the main causes of the degradation of the entanglement are multiple photons from the photon pair and the coherent light pulse, and mode mismatch at the quantum parity check. The ratios of both errors to the desired events are almost independent of the transmittance *T*. As a result, the DFS scheme will work well as long as the count rate is much larger than the dark count rate of the detectors.

**Figure 6:**
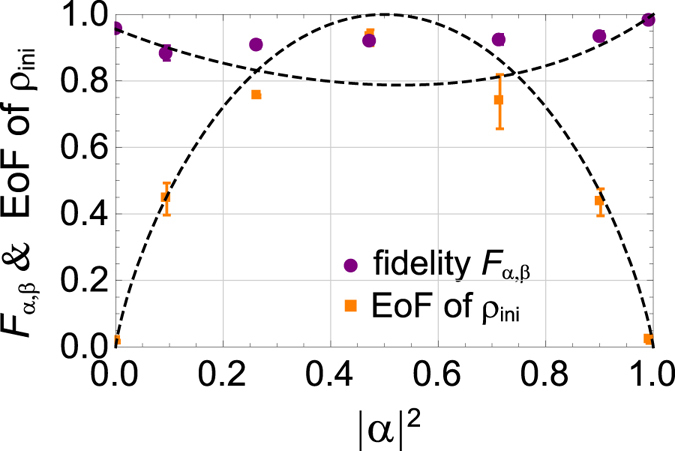
The observed fidelity *F*_*α*,*β*_ between *ρ*_ini_ and *ρ*_fin_ (circle), and the EoF of *ρ*_ini_ (square) for various values of |*α*|^2^ which is estimated from the probabilities of the component |HH〉 of *ρ*_ini_. A dashed curve for EoF is given by using the ideal state of |*ϕ*_*α*,*β*_〉. A dashed curve for the fidelity is given by the inner product between |*ϕ*_*α*,*β*_〉 and the final state with considering the multiple emission events and the mode matching effect.

Our method is applicable to the protection of not only the maximally entangled state but also a state in the form of |*ϕ*_*α*,*β*_〉 = *α*|HH〉 + *β*|VV〉, where *α* and *β* are arbitrary complex numbers satisfying |*α*|^2^ + |*β*|^2^ = 1. To see this, we prepared such non-maximally entangled states by rotating the polarization of the pump light, and then performed the DFS method. We reconstructed the initial and the final states, and calculated the fidelity between the two states. From the experimental result shown in Fig. 6, the fidelity ${F}_{\alpha ,\beta }={({\rm{tr}}|\sqrt{{\rho }_{{\rm{ini}}}}\sqrt{{\rho }_{{\rm{fin}}}}|)}^{2}$ between the observed initial state *ρ*_ini_ and the final state *ρ*_fin_ is larger than 0.84 for any value of |*α*|^2^. As a result, we see that our DFS method is useful for sharing the state |*ϕ*_*α*,*β*_〉. By using a quantum encorder^21^ based on the quantum parity check, any state in *α*|H〉 + *β*|V〉 can be encoded to the form of |*ϕ*_*α*,*β*_〉 without revealing the values of *α* and *β* at Alice’s side. Decoding can be performed by measuring the photon at Alice’s side by |D〉 after the DFS scheme with a use of a HWP, a PBS and a photon detector as 〈D||*ϕ*_*α*,*β*_〉 ∝ *α*|H〉 + *β*|V〉. This means that any single qubit can be sent from Alice to Bob by using the DFS scheme.

## Discussion

**Figure 7:**
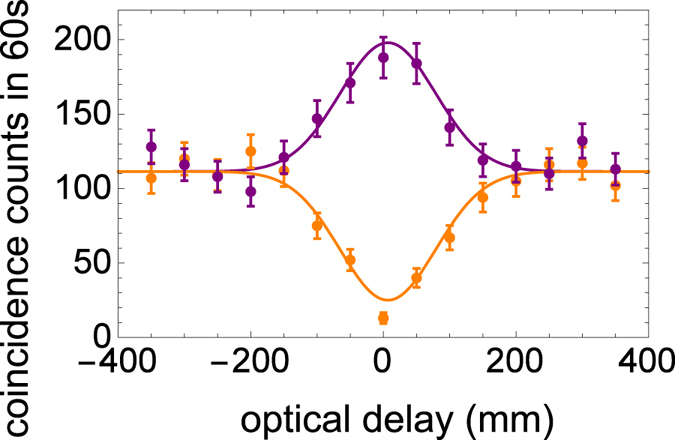
The two photon interference between the photon in mode A and the coherent light pulse R for *T* = 1.

The DFS scheme uses two interferometers, one of which is used for sending the photons by two quantum channels and the other is used for performing the quantum parity check at Alice’s side. In the former interferometer, while unknown phase shift between H and V may be added by the fluctuations of the two quantum channels, it is automatically canceled by the DFS. Thus, it is insensitive to the timing mismatch between the photons passing through the two arms^12^. The latter interferometer used for the quantum parity check is also insensitive to the timing mismatch between the photons from the SPDC and the coherent light pulse R because the two-photon interference^26^ is used. In fact, we did not perform any active stabilization of the two interferometers over 60 hours during our experiments. An observed dependency of the coincidence counts between the photon in mode A and the coherent light pulse R on the timing mismatch by moving mirror M at the quantum parity check in Fig. 2 is shown in Fig. 7. An observed full width at the half maximum is about ≈150 *μ*m which is much longer than the wavelength of the photons. As a result, the DFS method is totally robust against the fluctuations of the optical circuit, and does not need the control with the wavelength-order precision.

In conclusion, we have demonstrated the robust entanglement-sharing scheme over collective noisy channels. By using the counter-propagating reference classical light with an intensity inversely proportional to the transmittance *T* of the quantum channel, we experimentally achieved the entanglement-sharing rate scaling proportional to *T*. We also demonstrated that our method is used to distribute any state in *α*|HH〉 + *β*|VV〉, which indicates that it is applicable to distributing any unknown single qubit with the redundant encoding by the quantum parity check. The essence of the scheme is the use of the reciprocal property of the channel and the property of the entanglement that a disturbance of one half of the entangled system is equivalent to that of the other half. Since optical fibres are known to be reciprocal media and its fluctuations are slow enough to satisfy the collective assumption, we believe that our efficient DFS method will be useful to distribute entanglement for optical fibre communication over a long distance. In addition, it may open up a new sensor for detecting non-reciprocal property of the noisy channel by measuring the quantity of entanglement.

## References

[1] Gisin N, Ribordy G, Tittel W, Zbinden H (2002). Quantum cryptography. Rev. Mod. Phys..

[2] Lo H-K, Curty M, Tamaki K (2014). Secure quantum key distribution. Nature Photonics.

[3] Sangouard N, Simon C, De Riedmatten H, Gisin N (2011). Quantum repeaters based on atomic ensembles and linear optics. Reviews of Modern Physics.

[4] Cirac JI, Ekert AK, Huelga SF, Macchiavello C (1999). Distributed quantum computation over noisy channels. Phys. Rev. A.

[5] Broadbent, A., Fitzsimons, J. & Kashefi, E. Universal blind quantum computation. In *Foundations of Computer Science, 2009. FOCS’09. 50th Annual IEEE Symposium on*, 517–526 (IEEE, 2009).

[6] Lidar, D. A. & Whaley, K. B. Decoherence-free subspaces and subsystems. In *Irreversible Quantum Dynamics*, 83–120 (Springer, 2003).

[7] Kwiat PG, Berglund AJ, Altepeter JB, White AG (2000). Experimental verification of decoherence-free subspaces. Science.

[8] Walton ZD, Abouraddy AF, Sergienko AV, Saleh BEA, Teich MC (2003). Decoherence-free subspaces in quantum key distribution. Phys. Rev. Lett..

[9] Boileau J-C, Gottesman D, Laflamme R, Poulin D, Spekkens RW (2004). Robust polarization-based quantum key distribution over a collective-noise channel. Phys. Rev. Lett..

[10] Bourennane M (2004). Decoherence-free quantum information processing with four-photon entangled states. Phys. Rev. Lett..

[11] Boileau J-C, Laflamme R, Laforest M, Myers CR (2004). Robust quantum communication using a polarization-entangled photon pair. Phys. Rev. Lett..

[12] Yamamoto T, Shimamura J, Özdemir ŞK, Koashi M, Imoto N (2005). Faithful qubit distribution assisted by one additional qubit against collective noise. Phys. Rev. Lett..

[13] Chen T-Y (2006). Experimental quantum communication without a shared reference frame. Phys. Rev. Lett..

[14] Prevedel R (2007). Experimental demonstration of decoherence-free one-way information transfer. Phys. Rev. Lett..

[15] Yamamoto T (2007). Experimental ancilla-assisted qubit transmission against correlated noise using quantum parity checking. New Journal of Physics.

[16] Yamamoto T, Hayashi K, Özdemir ŞK, Koashi M, Imoto N (2008). Robust photonic entanglement distribution by state-independent encoding onto decoherence-free subspace. Nature Photonics.

[17] Takeuchi Y, Fujii K, Ikuta R, Yamamoto T, Imoto N (2016). Blind quantum computation over a collective-noise channel. Phys. Rev. A.

[18] Kempe J, Bacon D, Lidar DA, Whaley KB (2001). Theory of decoherence-free fault-tolerant universal quantum computation. Phys. Rev. A.

[19] Ikuta R (2011). Efficient decoherence-free entanglement distribution over lossy quantum channels. Phys. Rev. Lett..

[20] Kumagai H, Yamamoto T, Koashi M, Imoto N (2013). Robustness of quantum communication based on a decoherence-free subspace using a counter-propagating weak coherent light pulse. Phys. Rev. A.

[21] Pittman TB, Jacobs BC, Franson JD (2001). Probabilistic quantum logic operations using polarizing beam splitters. Phys. Rev. A.

[22] James DFV, Kwiat PG, Munro WJ, White AG (2001). Measurement of qubits. Phys. Rev. A.

[23] Řeháček J, Hradil Z, Knill E, Lvovsky AI (2007). Diluted maximum-likelihood algorithm for quantum tomography. Phys. Rev. A.

[24] Wootters WK (1998). Entanglement of formation of an arbitrary state of two qubits. Phys. Rev. Lett..

[25] Chuang IL, Nielsen MA (1997). Prescription for experimental determination of the dynamics of a quantum black box. Journal of Modern Optics.

[26] Hong C, Ou Z, Mandel L (1987). Measurement of subpicosecond time intervals between two photons by interference. Physical Review Letters.

